# 

**Published:** 1840-12

**Authors:** 


					﻿THE
WESTERN JOURNAL
OF
MEDICINE AND SURGERY.
COLLABORATORS.
Edward H. Barton, M. D. Profes-
sor of the Theory and Practice oj
Medicine, in the Medical College oj
Louisiana.
William J. Barbee, M. D. George
town, Ky.
George W. Bayless, M. D. Demon-
strator of Jinatomy in the Louis-
ville Medical Institute.
A- H. Buchanan, M. D. Columbia,
Tennessee.
Charles Caldwell, M. D. Professor
of the Institutes of Medicine and
Medical Jurisprudence in the Lou-
isville Medical Institute.
Samuel A. Cartwright, M D. Mis-
sissippi.
A. Clapp, M. D. New Albany, In-
diana.
Jedediah Cobb, M. D. Professor of
Anatomy in the Louisville Medical
Institute.
John Esten Cooke, M. D. Professor
of the Theory and Practice of Medi-
cine in the Louisville Medical In-
stitute.
Samuel D. Gross, M. D. Professor of
Surgery in the Louisville Medical
Institute.
John Hardin, M. D. Munfordsville,
Ky.
John P. Harrison, M. D. late Profes-
sor of Materia Medica, in the Cin-
cinnati College.
John F. Henry, M. D. Bloomington,
Illinois.
Samuel P. Hildreth, M. D. Marietta,
Ohio.
Samuel Hogg, M. D. Nashville, Ten-
nessee.
M. Z. Kreider, M. D., Lancaster,
Ohio.
Moses L Linton, M. D. Springfield,
Kentucky.
Henry Miller, M. D. Professor of
Obstetrics and the Diseases of Wo-
men and Children in the Louisville
Medical Institute.
John W. Monette, M. D. Washing-
ton, Mississsippi.
Samuel B. Richardson, M. D., Lec-
turer on Anatomy and Surgery, fyc,
Louisville, Ky.
John L. Riddell, M. D. Professor of
Chemistry in the Medical College
of Louisiana.
Landon C. Rives, M. D. late Pro-
fessor of Obstetrics in the Medical
Department of the Cincinnati Col-
lege.
Charles W. Short, M. D. Professor
of Materia Medica in the Louisville
Medical Institute.
G. Troost, M. D. Professor of Chem-
istry and Mineralogy in the Uni-
versity of Nashville
Amasa L. Trowbridge, M. D. Pro-
fessor of Surgery, Willoughby Uni-
versity, Ohio.
John A. Warder, M. D. Cincinnati,
Ohio.
William Wood, M. D. Cincinnati,
Ohio.
INDEX TO VOL. II.
A
Anatomy Pathological, Gross
on, -	131
Addison on the Disorders of
the Brain,	-	-	196
Ashwell on the Incision of
the Uterus,	-	-	127
American Journal	of Medical
Sciences,	-	-	-	428
Aneurism of the Aorta,	326
Acetate of Lead, Fatal Effects
of in large doses,	-	305
B
Bladder, Paralysis of,	-	34
Ballard’s Case of Removal of
the Uterus, -	-	156
Bayless’ Case of Testis in the
Abdomen, -	-	-	32
Bayless’ Hospital Reports, 325
Buchanan’s Case of Fracture
of the Skull,	-	-	96
Bicking on Poisoning by Ace-
tate of Lead,	-	-	305
Bird on Poisoning by Vapors
of Charcoal,	-	-	215
Buchanan on Negro Consump-
tion,	_	-	_	405
Bennett on Oil of Turpentine, 418
Bouchelle on the Medical Prop-
erties of the Cotton Plant, 162
C
Cancer, New Treatment of,	68
Cartwright on Medical Statis-
tics,	...	1
Crania Americana, 35,	-	105
Congenital Fungus Haemato-
des, .	_	_	.	79
Children, Partial Paralysis in, 82
Cranium, Feature of, with Loss
of Brain,	-	-	_	96
Cotton Plant, Medical Proper-
ties of,	-	-	.	162
Caldwell on	the Nervous Sys-
tem, -	-	-	.	165
Charcoal, Vapors of, A Case
of Poisoning by, -	220
Cataract,	- - _ 238
Camphor, its Effects on Veg-
etables,	_ _ _ 239
Catalogue of Kentucky Plants, 283
Calomel, its Transformation in-
to Corrosive Sublimate, 307
Cure of Strabismus, -	310
Corrosive Sublimate from Cal-
omel,	_	-	.	307
Cogley on Stramonium in Dys-
menorrhoea, -	-	338
Caloric, Sensibility to,	340
Cow-Pox, -	-	_	400
Consumption—Negro, -	405
D
Disorders of the Brain con-
nected with [Diseased Kid-
neys,	_	_	_	196
Dover’s Powder Modified, 316
Dysmenorrhosa, -	-	338
Dysentery, -	-	-	329
Dropsy,	...	333
Drake’s Case of Intermittent
Fever, .	-	_	345
Dennis on Phimosis and Para-
phimosis, -	-	-	393
Diseases of the Summer and
Autumn 1840,	-	-	399
E
Dashiell’s Case of Enlarged
Spleen, -	99
Emetics in Haemorrhage, -	309
Enlargement of the Spleen,	99
Eupatorium Perfoliatum, -	79
Elements of Pathological Anat-
omy,	_	_	.	131
“	“	-	187
“	“	-	289
“	“	-	367
F
Fever, Scarlet, -	-	90
Fever, Intermittent, '•	-	340
Fever, Bilious, a Premium for
Essay on, -	-	-	404
Fungus Hcmatodes,Congenital, 79
Fungus Hematodes, a case re-
sembling, cured, -	-	433
Fracture of the Cranium,	96
G
Green County Medical Society, 402
Gross on Pathological Anat-
omy, -	131
“	“	-	187
“	“	-	289
“	“	-	367
Guy’s Hospital Reports,	195
Guy on the Variation of the
Pulse, _	-	_	210
Gangrene of the Mouth, 422
H
Hospital Reports, Louisville, 325
Hospital Reports, Guy’s, 195
Hepatitis, -	-	_	340
Horner’s Necrological Notice
of Physick,	- -	393
Hemiphlegia,	- -	317
Hospital, Louisville Marine, 319
Health of Louisville, -	321
I
Introsusception cured by forc-
ing Air into the Intestines, 57
Iron in Anemic Diseases, 159
Imperforate Uterus, -	224
Incision in Cases of Occlusion
of the Uterus, -	-	227
Institute, Medical, Louisville, 404
Institute Medical, Lancaster, 315
Intermittent Fever, -	340
Ipecacuanha in Hemorrhage, 309
Inhalation of Carburetted Hy-
drogen, _	_	.	220
K
Kidneys, Disease of Affecting
the Brain,	-	-	196
L
Lawrie on Scarlet Fever,	90
Linton on Medicine in Paris, 69
“	“	“	159
Lobelia in Sick-Headache, 78
Lead, Acetate of, Poisoning by, 305
Laryngitis, -	340
Licking County Medical So-
ciety,	_	_	_	401
Lancaster Medical Institute, 315
Lindsy’s Introductory, -	484
M
Medical Statistics of Natchez, 1
Monette on Sulphate of Qui-
nine, -	-	-	-	21
Morton on Crania Americana, 35
“	“	•<	105
Monstrosity, a Case of,	-	61
Medicine in Paris,	-	69
“	“	159
Milk-Sickness, -	-	80
“ “ - . . 101
Medical Miscellany, -	84
Medical Properties of the Cot-
ton Plant,	-	-	162
Menorrhagia, Monesia in,	231
Medical Convention at Frank-
fort, -	-	-	-	481
Medical Obituary, •-	-	481
Monesia in Menorrhagia, 231
Mouth, Gangrene of,	- 422
N
Natchez, Medical Statistics of, 1
Neuralgia Cured by the Extrac-
tion of a Tooth,	-	335
Negro Consumption,	-	405
Natchez Tornado,	-	74
Nervous System, Influence of, 165
Nasal Polypus Cured by San-
guinaria Canadensis, -	237
0
Observations on Poisoning by
Burning Charcoal, -	215
Occlusion of the Uterus, Incis-
ion for, -	-	-	227
Oil of Turpentine,	-	418
P
Payne’s Commentaries,	484
Physick, Memoir of, -	353
Pneumonitis,	- - 340
Pathological Anatomy, Gross’
Elements,	-	-	131
“	“	-	367
Phimosis and Paraphimosis,
Ricord on,	-	-	391
Philosophical Society, Ameri-
can,	_	_	-	401
Perrine, Necrological Notice
of, -	-	-	-	321
Perforations of the Stomach, 201.
Poisoning by inhalation of Car-
buretted, Hydrogen, -	220
Poisoning by Vapors of Char-
coal,	-	-	-	215
Paralysis of the Bladder,	34
Paralysis, partial in Children, 82
Prevention of Tubercles,	50
Paris, Medicine in,	-	67
“	“	159
Pulse, Variations of,	- 210
Pin retained in body nineteen
years, and then discharged, 402
Q
Quinine, Sulphate of in Fevers,
its action, -	-	-	21
R
Randolph’s Memoir of Plys-
ick, -	-	-	-	----
Rectus Muscle, its Division for
Strabismus, -	-	58
Reports Hospital, Guy’s, 195
Rigidity of the Uterus, Incis-
ion for. -	-	-	227
Richardson on Tenotomy, 245
Ricord on Phimosis and Para-
phimosis, -	_	_	393
Rain, Quantity of for nine
years, -	396
Review of Gross’ Elements, a
Reply to, -	-	-	425
Removal of the Uterus by
force, -	156
Reports Hospital, Louisville, 325
Red Sulphur Springs, -	310
S
Statistics Medical of Natchez, 1
Sulphate of Quinine, -	21
Sulphate of Quinine, adultera-
tion of, _	-	-	60
Scrofulous Diseases,Oil of Cod-
fish in, -	-	-	67
Syphilis, Treatment of,	-	67
Sick Headache, lobelia in, 78
Sick-Stomach,	-	-	89
Spina Bifida, -	-	-	85
Scarlet Fever,	-	-	95
Spleen, Chronic enlargement of, 99
Stramonium in Trismus, 239
Strabismus, Surgical Cure of, 310
Sanguinaria Canadensis in Na-
sal Polypus, -	-	237
Supplementary Catalogue of
Kentucky Plants, -	283
Short’s Catalogue of Kentucky
Plants, -	-	-	283
Sick-Headache, tea and coffee
as the cause of, -	-	320
Surgery Operative, Velpeau’s, 321
Stramonium in Dysmenorrhoea, 338
Scarlet Fever, -	-	400
Society Medical of Tennessee, 404
Strabismus, its Cure, -	475
T
Tenotomy, its History, and
Mode, _	_	_	245
Temporary Hemiplegia, -	317
Temperance Reform, its Pro-
gress, -	-	-	318
Transactions of the Medical
Society of New York, 382
Townsend on Sensibility to Ca-
loric, -	-	-	-	340
Tincture of Eupatorium Per-
foliatum,	-	-	-	79
Tornado Natchez, -	-	74
Tubercles Prevention of,	60
Travis on Milk-Sickness,	101
Trismus Stramonium in,	239
Testis in the Abdomen, -	32
Todd’s Case of Paralysis of
the Bladder, -	-	24
Trowbridge on Spina Bifida, 85
Turpentine, Oil of, -	418
U
Uterus, Forcible Removal of,
Treatment, -	-	156
Uterus, Incision of in Cases
of Occlusion, -	-	224
Uterus Imperforate,	-	227
V
Vaccination, Value of,	-	65
Vapors of Burning Charcoal,
Poisoning by, -	-	215
Vegetables, Effects of Camphor
on, -	-	-	-	249
Velpeau’s Operative Surgery, 321
□CFThe subscription year of the Western Journal
of Medicine and Surgery is now ended, and we
have now a right to call upon subscribers, one and
all, to remit. Money enclosed to us by mail is always
at our risk, but subscribers are expected to procure
the post office frank or to pay postage.
PRENTICE & WEISSINGER.
Louisville, Nov. 2d, 1840.
The Western Journaj, of Mf.dtcj\e and^SuRGERY is published monthly by
the undersigned, at the,cornermf Main and Fifth streets, Louisville, at $5 per
annum, payabledn ^vance^rlfftch^n^mher contains from 80 to 84 pages making
two volumes in the year of.abouttfllO-pagtjs.
Contribution® to its^pasres by fhp PhvsiWahs-of the Valley of the Mississippi,
addressed to thaEditors, areu-esj^^jjfcjj^solicited.
Letters on bnsmtess>tQ» be addressed,’pAsta^e paid, to the publishers.
July 25, 1840.	PRENTICE & WEISSINGER
				

